# TGF-β2 and KL-6 as potential biomarkers for hard metal lung disease

**DOI:** 10.1186/s12890-026-04173-7

**Published:** 2026-02-14

**Authors:** Zhansai Zhang, Qian Yao, Yanfang Zhao, Kongrong Guo, Jinbo Zhang

**Affiliations:** 1 Department of Occupational Disease, Shanghai Pulmonary Hospital, Tongji University School of Medicine, Shanghai, 200433 China; 2 Clinical Research Administration Department, Shanghai Pulmonary Hospital, Tongji University School of Medicine, Shanghai, China; 3 Clinical Research and Development Center of Shanghai Municipal Hospitals, Shanghai Hospital Development Center, Shanghai, China; 4 Department of Military Health Statistics, Naval Military Medical University, Shanghai, China

**Keywords:** Hard metal lung disease, Pulmonary fibrosis, Cytokine, BALF, Biomarker, Inflammation

## Abstract

**Backgrounds:**

Hard metal lung disease (HMLD) is an occupational disease caused by exposure to hard metal (HM) dust. We explored TGF-β1, TGF-β2 and KL-6 as potential biomarkers for HMLD by examining their levels in bronchoalveolar lavage fluid (BALF) and serum.

**Methods:**

Forty-eight female Sprague-Dawley rats were divided into hard metal (HM) and normal control (NC) groups. The HM group was exposed to tungsten carbide-cobalt dust (WC-Co) dust via intratracheal instillation. Cytokine levels in BALF and serum were measured at 4-, 8-, and 12-weeks. Lung changes were assessed by micro-CT and histopathology.

**Results:**

At 4-, 8-, and 12-weeks, the micro-CT showed ground glass opacities, diffuse ground-glass nodules, and scattered ground glass opacities in both lungs of HM group rats, while the pathological tissue found inflammatory cell infiltration, a large number of multinucleated giant cells, and inflammatory cell infiltration, respectively. TGF-β2 levels in BALF were significantly higher in the HM group at 8 and 12 weeks, while serum TGF-β2 levels were lower. KL-6 levels in BALF were elevated in the HM group at 12 weeks, but serum KL-6 levels were lower at 8 weeks.

**Conclusions:**

TGF-β2 and KL-6 levels in BALF and serum demonstrate dynamic changes associated with HMLD progression and may serve as potential biomarkers for diagnosis and monitoring. Further studies are needed to explore the underlying mechanisms.

## Introduction

Hard metal lung disease (HMLD) is a kind of occupational disease caused by the repeated inhalation of carbide dust (e.g. tungsten, titanium, etc.) over many years, characterized by interstitial giant cell pneumonia [27]. HMLD can occur about a year after contact with solid metals, but usually develops 10 to 20 years after exposure. Among workers exposed to solid metals, the incidence of HMLD ranged from approximately 0.13% to 3.8% [19]. In the early stages of HMLD, symptoms such as coughing and difficulty breathing gradually decrease, lung function is gradually restored, and abnormal lung imaging findings improve. However, if exposure continues, the disease can relapse and worsen. Repeated contact sensitization may lead to irreversible lung interstitial fibrosis [11, 23].

The early imaging manifestations are ground-glass consolidation shadows and diffuse nodules in both lungs, while the late manifestations include extensive reticular cystic shadows and traction bronchiectasis [7]. Examination of pulmonary function reportedly shows restrictive ventilation dysfunction and decreased diffusion function, as well as decreased FEV1 and FEV1/FVC indicators [10]. Individuals exposed to cemented carbide for prolonged periods may develop occupational asthma, which is similar to allergic pneumonia (hypersensitivity pneumonitis), and interstitial lung disease [13, 16, 20].

Hard alloy is a special type of alloy based on tungsten carbide and cobalt as the bonding phase. The content of tungsten in its composition is usually between 70% and 95%, while cobalt accounts for 5% to 30%, playing a role as a binder matrix [18]. Tungsten carbide cobalt (WC-Co) is irreplaceable in various industries, owing to its hardness, high bending strength, and strong wear resistance [25]. WC-Co dust is released into the air through the extensive and repeated use of tools, such as drills, in a closed environment. Inhalation of WC-Co dust is known to cause occupational asthma and HMLD and increases the risk of lung cancer twofold [2]. However, the relationship between the exposure, toxicity, and disease development remains poorly understood. Among pulmonary diseases, HMLD is difficult to diagnose as its symptoms are similar to those of other respiratory ailments.

The pathological manifestation of HMLD is often multinucleated giant cell central lobular fibrosis, resulting in giant cell interstitial pneumonia [1, 6, 8]. Herein, we used data and images from a previous experiment to hypothesize that various cytokines play vital roles in HMLD initiation and progression. Transforming growth factor-β1 (TGF-β1) is a valuable biomarker for HMLD diagnosis. The experimental results obtained in week 8 of this study are from a previously published paper [28]. However, the experimental results for weeks 4 and 12 have not yet been published. We aimed to establish and improve a rat model of HMLD to observe the dynamic changes in TGF-β1, TGF-β2, and Krebs von den Lungen-6 (KL-6) levels in the serum and BALF and investigate the effects of HMLD on TGF-β1, TGF-β2, and KL-6. These findings may elucidate the toxic effects and help identify potential biomarkers for the diagnosis and monitoring of HMLD.

## Methods

Forty-eight adult female Sprague-Dawley rats (weighing 190 ± 10 g) were purchased from Shanghai Shrek HM Animal Co., Ltd. (2012-0002; laboratory animal production license number SCXK; Shanghai, China). The rats were housed in an air-conditioned room at 25 °C, with controlled humidity, and a 12-h light/dark cycle; food and water were provided *ad libitum*. The rats were acclimated for at least two days before use. The rats were randomly divided into two groups with 24 animals per group. The animal experiment was approved by the Ethics Review Committee of the Animal Research Institution of Tongji University (K15-033). All efforts were made to ensure minimal suffering for the rats during the procedures.

Hard metal (HM) powder (WC-Co particles, tungsten carbide-cobalt mixed powder: tungsten carbide 80%, cobalt 20%) was purchased from a cemented carbide manufacturing enterprise in Shanghai, China. All reagents were suspended in normal saline at a concentration of 20 mg/mL and used as test substances. The rats were randomly divided into experimental (*n* = 24, HM group) and control groups (*n* = 24, normal control [NC] group). Based on preliminary dose-response experiments, an exposure dose of 10 mg per animal was selected. After exposure for 4, 8, and 12 weeks, eight animals in each group were euthanized.

Most in vivo investigations to assess the lung toxicity of cobalt and related powders have been performed using intratracheal instillation. Therefore, the HM stock solutions were sonicated for 5 min on ice to ensure particle dispersion and used immediately for intratracheal instillation. The rats were anesthetized with thiobutabarbital sodium salt hydrate (Inactin; Sigma-Aldrich, St. Louis, MO, USA) at a dose of 1 mg/kg via intra-peritoneal injection. After anesthesia, an aerosolizing micro sprayer was used to administer 0.5 mL of the aerosolized test substance to the HM group rats and normal saline to the NC group rats, via the trachea. The rats were monitored after instillation until consciousness was regained.

Anesthesia was confirmed by testing the toe-pinch reflex. Upon euthanizing, blood from the rats was collected into vacuum tubes via cardiac puncture, and a minimum of 6 mL of blood was obtained. The blood was allowed to clot and centrifuged at 1000 × *g* for 10 min at 20 °C to obtain the serum. The supernatant was collected and stored at -80 °C until analysis. Bronchoalveolar lavage fluid (BALF) was acquired by gently flushing the left lung three times through the trachea with 1 mL of 0.9% saline and pre-warmed to 37 °C. BALF (2 mL) was recovered from each rat and centrifuged at 1000 × *g* for 10 min at 20 °C. The supernatant was collected and stored at -80 °C until analysis.

TGF-β1 and TGF-β2 levels in the serum and BALF were assayed using ELISA kits (R&D Systems; Minneapolis, MN, USA). KL-6 levels in the serum and BALF were assayed using kits from Cloud-Clone (Houston, TX, USA). Serum samples from each group and the antigen standard were added to the corresponding wells of the reagent plate, followed by biotinylated antibody working solution. A plate-sealing film was used to seal the microplate wells, and the plate was incubated for 2 h at 25–30 °C. The plate was rinsed four times using an automated microplate washer. Next, the enzyme-conjugated working solution was added to the wells; the plate was sealed and incubated for 30 min at 25–30 °C, and then rinsed four times. Substrates A and B were added into each well of the plate. An ELx800 automatic microplate reader (BioTek, Winooski, VT, USA) was used to measure the absorbance of the samples at 450 nm. Each sample was measured twice, and the average value was recorded. A standard curve was constructed and used to calculate the cytokine levels in each sample. The BALF was analyzed similarly.

After treatment for 4, 8, and 12 weeks, the rats were evaluated using micro-computed tomography (micro-CT) and histopathological examinations. After anesthetizing the rats with the respiratory anesthesia equipment, the eXplore Locus micro-CT system (GE Healthcare, Little Chalfont, UK) was used for small animal tomography, with a scan range from the apex of the lungs to the upper abdomen. The scanning parameters were as follows: voltage, 50 kV; current, 450 mA; exposure time, 300 ms; scan technique, 360; detector bin mode, 4⋅4; frames averaged, two; scan time, 16 min; and spatial resolution, 0.092 mm. Microview software (version number 10.01) built-in with the system was used for image processing.

Obtain lung specimens from rats under continuous anesthesia and mechanical ventilation. After disinfection, open the chest to expose the lungs. Remove the tissue from the middle lobe of the right lung, fix it in a 10% formaldehyde solution, embed it in paraffin, cut it into 5–10 µ m sections, and stain them with hematoxylin and eosin. Observe the histopathological changes of lung tissue using an optical microscope.

For the final sacrifice of the animals, we used carbon dioxide (CO₂) euthanasia, which is a widely accepted and ethically compliant method. The rats were placed in a chamber that was gradually filled with CO₂ at a flow rate of 10%-30% of the chamber volume per minute.

SPSS Statistics 22.0 software (SPSS Inc., Chicago, IL, USA) was used for data analyses. Data are expressed as the mean ± standard deviation. Comparison of data at different times between the NC and HM groups was performed using a two-way analysis of variance (ANOVA) and Bonferroni’s method of multiple comparison when there were effects of interaction between the HM factors and time points. The assumptions of two-way ANOVA including normal distribution and homogeneity of variance have been verified by the Shapiro-Wilk test (normality) and the Levene test (homogeneity of variance). In addition, there were no missing data in the formal experiments of this study, and no rats died due to accidents during the experiments. All tests were two-sided, and a two-tailed *P*-value ≤ 0.05 was considered to indicate statistical significance.

## Results

**Fig. 1:**
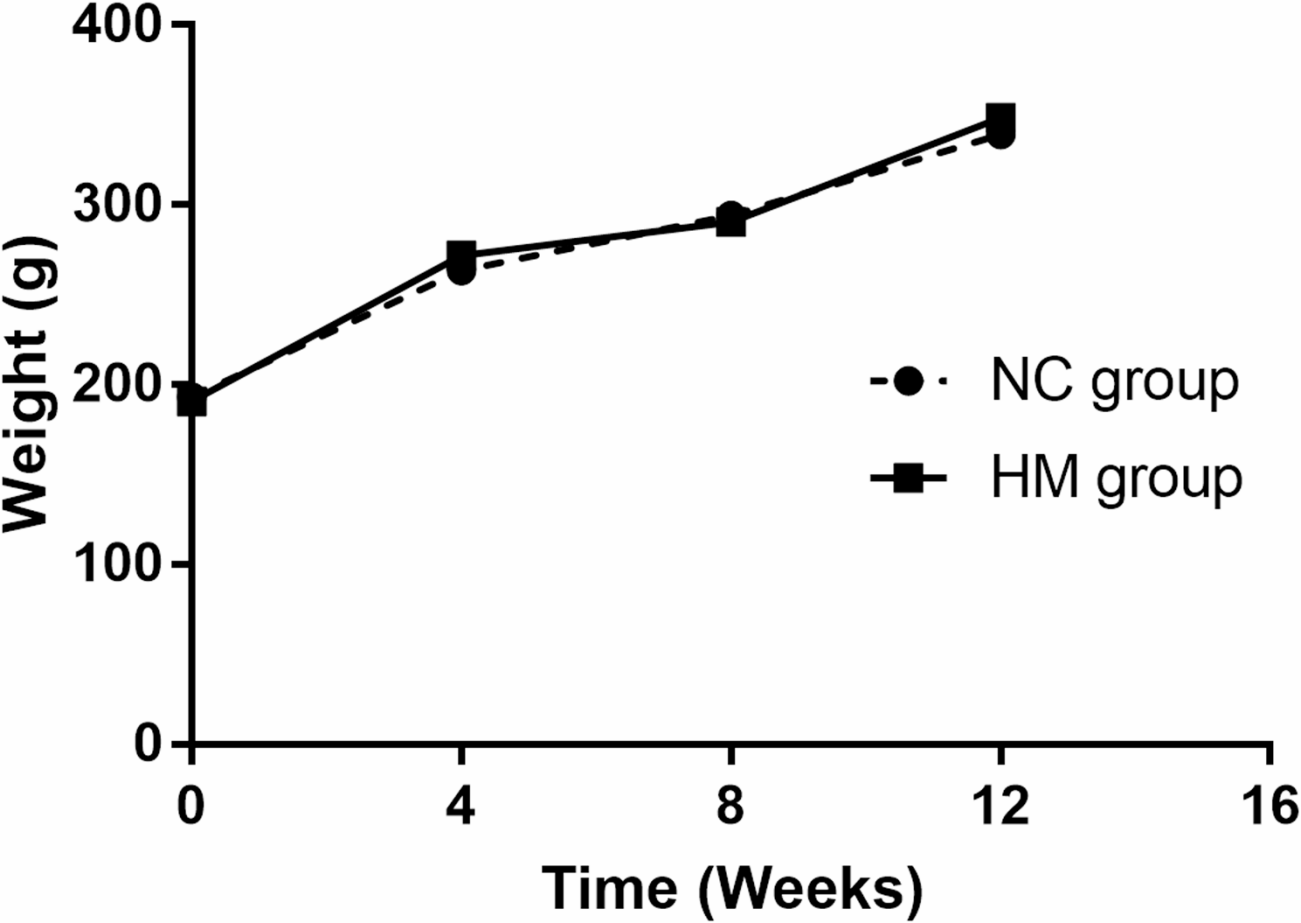
The trend of the weight change in HM exposed group and healthy control group of rats at 4, 8, and 12 weeks

The average body weight of rats in the two groups showed an increasing trend but there was no significant difference at the beginning of treatment and at 4, 8, and 12 weeks (Fig. 1).

**Fig. 2:**
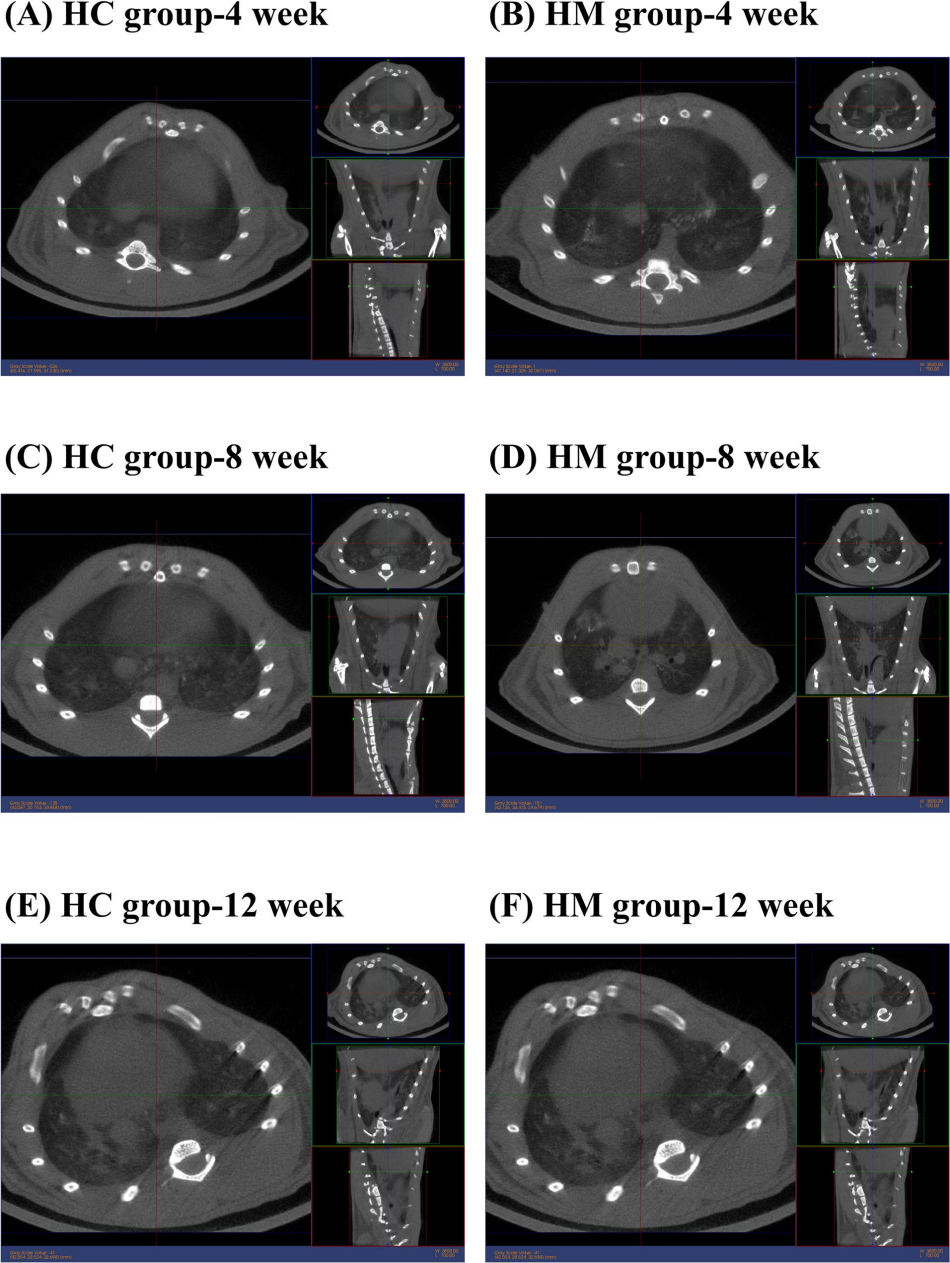
Pulmonary imaging of the rat lungs at different stages. **A** healthy control group rats at 4 weeks; (**B**) HM group rats at 4 weeks; (**C**) healthy control group rats at 8 weeks; (**D**) HM group rats at 8 weeks; (**E**) healthy control group rats at 12 weeks; (**F**) HM group rats at 12 weeks

**Fig. 3:**
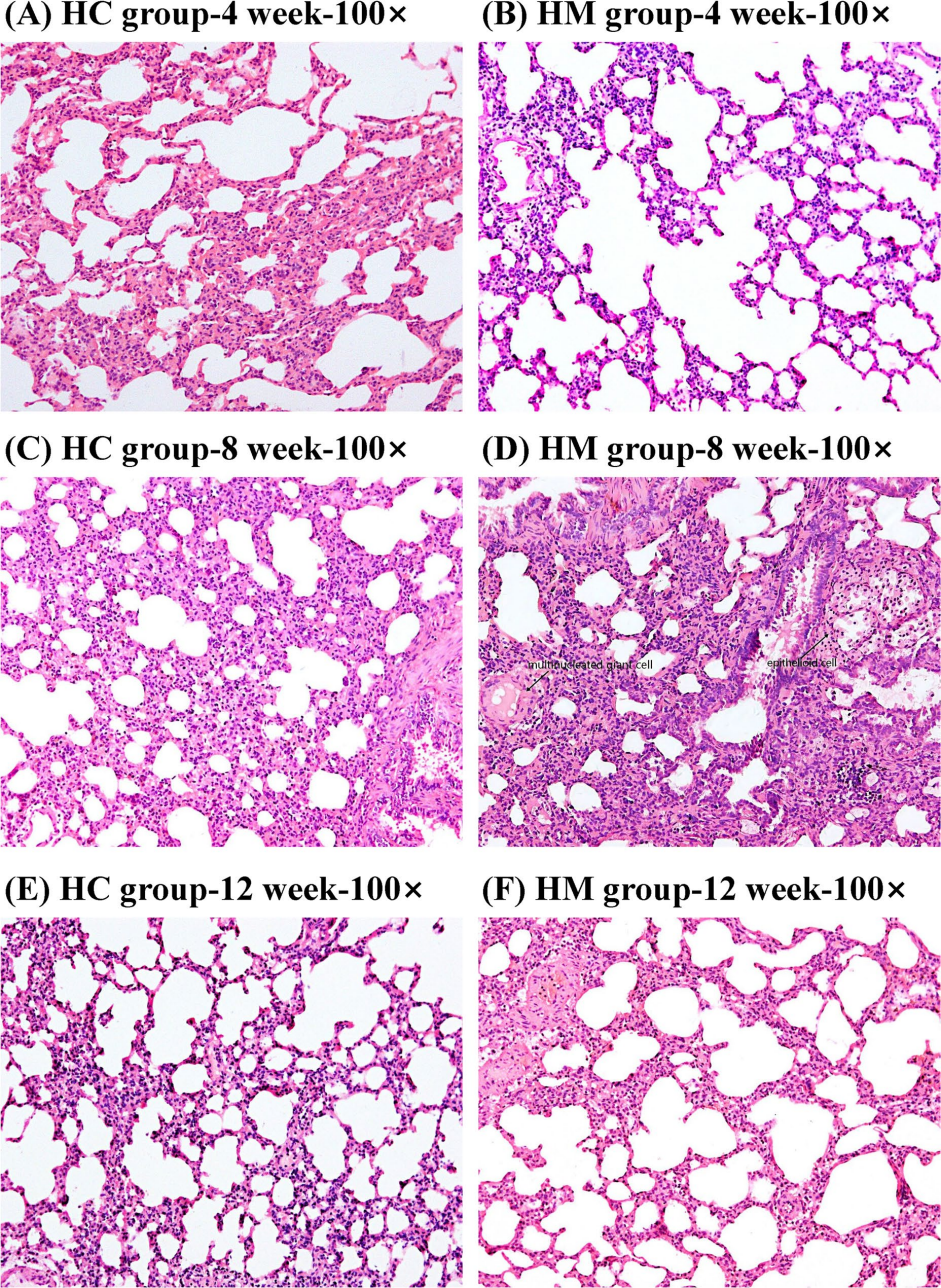
Histopathological changes in the rat lungs at different stages. **A**, (**C**), (**E**): healthy control group rats at 4, 8, 12 weeks; (**B**) 4 weeks: inflammatory cell infiltration in HM group of rats; (**D**) 8 weeks: appearance of multinucleated giant cells and epithelioid cells in HM group of rats; (**F**) 12 weeks: inflammatory cell infiltration in HM group of rats

At 4 weeks after exposure, compared with the NC group, the bilateral lungs of the HM group rats showed ground glass opacities. After 8 weeks, the bilateral lungs of HM group rats showed diffuse ground-glass nodules, accompanied by central lobular nodules, focal consolidation, extensive reticular and linear shadows. After 12 weeks, scattered ground glass opacities appeared in both lungs of HM group rats, and the abnormal shadows were not obvious (Figs. 2 and 3).

**Fig. 4:**
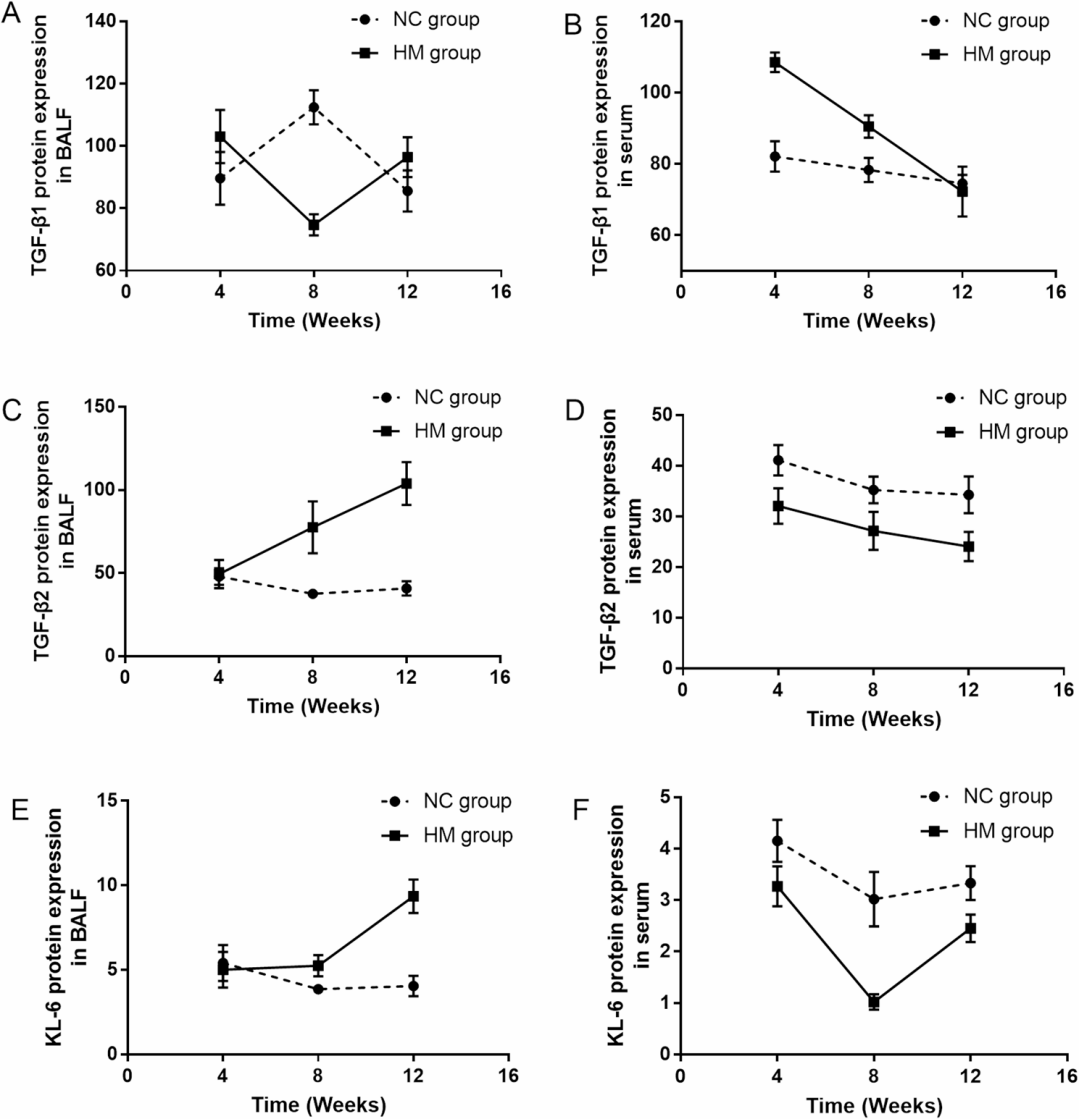
The comparison of TGF-β1, TGF-β2, KL-6 levels in BALF or serum among HM exposure group and healthy control group of rats at 4, 8, and 12 weeks using two-way ANOVA followed by Bonferroni post-hoc test (*P < 0.05, **P < 0.01 vs. control group). **A** TGF-β1 in BALF (**B**) TGF-β2 in serum (**C**) TGF-β2 in BALF (**D**) TGF-β2 in serum (**E**) KL-6 in BALF (**F**) KL-6 in serum

In the BALF, TGF-β1 level after 8-week exposure was significantly lower in the HM group (*P* = 0.009) while no significant difference was observed at 4 or 12 weeks. In the serum, however, 4-week TGF-β1 level in the HM group was significantly higher than that in the NC group (*P* = 0.002) while there was no significant difference at 8 or 12 weeks. The interaction between exposure and exposure time for TGF-β1 was significant in both the BALF (*P* = 0.001) and the serum (*P* = 0.009) (Fig. 4A and B).

For TGF-β2 level in the BALF, both the interaction between exposure and exposure time and the main effect of exposure was significant. There was a significant increase at 12 weeks after treatment in the HM group. However, there was no significant difference between the groups at the other time points (Fig. 4C). In the contrast, the serum TGF-β2 level in the HM group was significantly lower than that in the NC group (*P* = 0.003). The exposure time showed no effect on the serum TGF-β2 level (*P* = 0.100) and the interaction between exposure and exposure time was also not significant *(P* = 0.954) (Fig. 4D).

The KL-6 level in the BALF of HM rats at 12 weeks was higher than that of rats in the NC group (*P* = 0.001) while there was no significant difference between the groups at 4 or 8 weeks (Fig. 4E). The interaction between exposure and exposure time was significant (*P* = 0.007). The serum KL-6 level in the HM rats was lower than that in the NC rats (*P* < 0.001) at 8 weeks. The exposure time affected the serum KL-6 level (*P* < 0.001); however, there was no significant interaction between exposure and exposure time (*P* = 0.266) (Fig. 4F).

## Discussion

We compared the effects of exposure on two groups of rats (HM and NC) at 4, 8, and 12 weeks, focusing on body weight, pulmonary imaging, cellular changes, and cytokine levels in BALF and serum to investigate the dynamic changes of TGF-β1, TGF-β2 and KL-6 levels. Body weight increased similarly in both groups with no significant differences. Pulmonary imaging showed progressive changes in the HM group including ground-glass opacities at 4 weeks, diffuse nodules and other abnormalities at 8 weeks, and scattered opacities at 12 weeks. Cellular analysis revealed inflammatory cell infiltration at 4 weeks, multinucleated giant cells at 8 weeks, and further inflammation at 12 weeks. At 4 weeks, serum TGF-β1 level significantly increased. At 8 weeks, BALF TGF-β1 and serum KL-6 level was significantly lower. At 12 weeks, BALF TGF-β2 and KL-6 level increased while the serum TGF-β2 level decreased significantly.

HMLD often manifests as varying degrees of interstitial lung disease and pulmonary fibrosis, while interstitial pneumonia and pulmonary fibrosis caused by HM deposition are often accompanied by persistent low-grade inflammation, leading to imbalances in growth factors, cytokines, and tissue proteases [14]. After heavy metal (HM) dust enters the alveoli, it promotes the activation of alveolar macrophages. Activated alveolar macrophages aggregate and fuse into multinucleated giant cells, which induce inflammatory changes in the alveoli by secreting various inflammatory mediators [5]. HM dust can promote the proliferation of fibroblasts and ultimately cause inflammatory changes associated with pulmonary interstitial fibrosis.

The pathological features of HMLD include interstitial pneumonia of giant cells, characterized by the accumulation of macrophages and a large number of multinucleated giant cells in the alveolar cavity [21]. Multinucleated giant cells in lung tissue are specific markers for diagnosing HMLD and the most important histological markers for giant cell interstitial pneumonia [9]. The BALF characteristics of typical HMLD include an increase in total cell count, mainly macrophages, accompanied by a quantity of multinucleated cells [9].

TGF-β plays an important role in promoting the development of pulmonary fibrosis by promoting fibroblast proliferation and reducing collagen degradation [17]. In addition, it can stimulate fibroblast proliferation and promote the synthesis of extracellular matrix components such as collagen and fibronectin [26]. However, TGF-β is a metalloprotease α-2 that promotes the secretion of tissue inhibitors such as cell globulin, and inhibits the production of collagen and protease to reduce collagen breakdown [3, 22]. BALF Cytokine levels are associated with the development of pulmonary fibrosis [12]. This study found that the level of TGF-β1 in the serum of the HM group was lower than that of the NC group at 8 weeks after treatment, and the level of TGF-β1 in the serum of the HM group was higher than that of the NC group at 4 weeks after treatment, indicating that the level of TGF-β1 increased in BALF and decreased in serum. This is probably due to the increased permeability of alveolar capillaries by TGF-β1, as well as the high expression of TGF-β1 in the periphery of the body, indirectly indicating that HMLD can induce systemic inflammatory response at this stage, and it is no longer limited to the lungs. At 12 weeks, there was no difference in TGF-β1 levels between alveoli and serum; Therefore, TGF-β1 should not be used as a biomarker for the diagnosis and monitoring of HMLD. We found that the levels of TGF-β2 in BALF of HM group were higher than those of NC group at 8 and 12 weeks after treatment, and the levels of TGF-β2 in serum of HM group were lower than those of NC group at 8 and 12 weeks after treatment. This indicates that TGF-β2 can be used as a biomarker for the diagnosis and monitoring of HMLD.

KL-6 is a type II alveolar surface antigen, which is a chemotactic factor secreted by fibroblasts and has a chemotactic effect on them [4]. The serum KL-6 level of lung disease patients is elevated and accompanied by basement membrane damage, as this releases KL-6 into the alveolar folds and blood, leading to an increase in KL-6 levels in the blood. The KL-6 level in patients with pulmonary fibrosis decreases with the progression of pulmonary fibrosis [15, 24], whereas the KL-6 level in the BALF continues to increase [29]. The results showed that the KL-6 level in the serum of HM rats was lower than that of NC rats, and at 12 weeks, the KL-6 level in the BALF of HM rats was higher than that of NC rats. This indicates that KL-6 can be used as a biomarker for the diagnosis and monitoring of HMLD.

So far, since the research on HMLD is still limited, as far as we know, similar BALF-serum dissociations of TGF-β2 and KL-6 have not been reported. Therefore, we aimed to propose that in clinical practice, BALF and serum samples can be collected simultaneously during diagnostic bronchoscopy for patients with suspected HMLD to evaluate the relationship between the levels of TGF-β2 and KL-6. For confirmed patients, the progression of the disease can be indirectly reflected through regular monitoring of serum markers and combined with imaging examinations, making up for the limitations of repeated BALF tests.

This study found increased TGF-β2 and KL-6 levels in BALF alongside decreased serum levels at certain time points. Based on clinical experience, we have put forward three possible hypotheses. First, the core of HMLD damage is local inflammation and fibrosis in the lungs. TGF-β2 and KL-6 are mainly secreted by local epithelial cells and macrophages in the lungs. During the active phase of the disease (such as at 4 weeks and 12 weeks), they are preferentially enriched in lung tissue and BALF, forming a local high-concentration “vessel-like” state; Second, at 8 weeks, the lung injury is the most severe. The permeability of pulmonary capillaries significantly increases, which may cause some markers in the serum to migrate or leak to the local area of the lungs, while local inflammatory cells consume a large number of free markers in the serum, resulting in a decrease in serum levels; At 12 weeks, lung injury begins to repair, and the permeability of the lung barrier gradually recovers. The locally secreted markers are difficult to enter the serum, further exacerbating the trend of separation between BALF and serum. Third, combined with the pathological results, the increase in TGF-β2 and KL-6 in BALF is positively correlated with the degree of lung fibrosis (the fibrosis score at 12 weeks is higher than that at 8 weeks), while the decrease in serum levels may reflect that the disease is in the stage of local repair, and the systemic inflammatory response is weakened.

In this study, BALF TGF-β1 increased at 4 weeks and decreased at 8 weeks, which was inconsistent with the peak of lung injury (at 8 weeks). This might be related to the unique pathological process of HMLD. TGF-β1 mainly mediates inflammatory responses in the early stage of acute lung injury (1–4 weeks), while in the later stage of injury (after 8 weeks), it may be replaced by other fibrosis-related cytokines (such as TGF-β2).

At 12 weeks, micro-CT showed partial alleviation of lung injury, but TGF-β2 and KL-6 in BALF increased. This phenomenon might reflect the transition of the disease from the inflammatory injury stage to the fibrosis repair stage. As we all know, TGF-β2 is a key fibrosis regulatory factor, and during the injury repair stage, local fibroblasts in the lung are activated and secrete a large amount of TGF-β2, promoting collagen deposition; KL-6 is mainly secreted by type II alveolar epithelial cells during the injury repair process, and its increase suggests epithelial cell regeneration and remodeling.

This study provides a comprehensive analysis of multiple potential biomarkers (TGF-β1, TGF-β2, and KL-6) in BALF and serum. This dual approach allows for a more holistic understanding of the biological changes associated with HMLD and enhances the reliability of the findings. The study employs a longitudinal design, examining the effects of hard metal exposure over 4, 8, and 12 weeks. This time-course analysis helps elucidate the dynamic changes in biomarker levels and provides insights into the progression of HMLD, which is valuable for understanding disease development and potential therapeutic windows.

There are a few limitations in this study. First, the study is entirely based on an animal model without corresponding human data. While animal studies are crucial for initial hypothesis testing, validating these findings in human cohorts is necessary to confirm the biomarkers’ utility in clinical practice. Second, the study focuses on TGF-β1, TGF-β2, and KL-6, but other cytokines and biomarkers may also play important roles in HMLD. A broader panel of biomarkers could provide a more comprehensive understanding of the disease’s pathogenesis and identify additional diagnostic or prognostic markers. Third, the study demonstrates changes in biomarker levels but does not delve into the underlying molecular mechanisms driving these changes. Further research is needed to elucidate how hard metal exposure leads to alterations in TGF-β2 and KL-6 levels and how these changes contribute to the development of pulmonary fibrosis and inflammation in HMLD. Fourth, the experimental results obtained at 8 weeks have been published previously [28] in Chinese. However, the experimental results for weeks 4 and 12 have not yet been reported and this study provided clearer evidence of cytokine level change in different time point. Our previous Chinese-published study have reported the lung tissue pathological score, semi-quantitative score of micro-CT imaging, and serum TGF-β1 level at 8 weeks, therefore mainly focused on the single 8-week timepoint. In the current study, we have further explored the dynamic levels of TGF-β1, TGF-β2, and KL-6 in BALF at 4 weeks, 8 weeks, and 12 weeks, so that the levels of TGF-β1, TGF-β2, and KL-6 in serum and BALF were further reported at 4 weeks and 12 weeks as well as the dynamic correlation analysis with different disease stages (injury, peak, and repair) of all these three markers in BALF and serum. The new findings clarify the complete longitudinal dynamic monitoring and BALF-serum paired analysis.

## Conclusion

In summary, we successfully established and improved the HMLD rat model, and evaluated the levels of various cytokines associated with the disease. Our research suggests that the dynamic changes in TGF-β2 and KL-6 levels are potential biomarkers for diagnosing and monitoring HMLD. However, the potential molecular mechanisms need further investigation.

## Data Availability

The datasets used for this study are available from the corresponding author upon reasonable request.
